# Population-level associations of achievement of targets for bone-mineral markers with survival in haemodialysis patients with mildly elevated intact-PTH levels: a case-cohort study

**DOI:** 10.1038/s41598-019-47852-8

**Published:** 2019-08-05

**Authors:** Shingo Fukuma, Shunichi Fukuhara, Sayaka Shimizu, Tadao Akizawa, Masafumi Fukagawa

**Affiliations:** 1 Human Health Sciences, Kyoto University Graduate School of Medicine, Kyoto, Japan; 2 Department of Healthcare Epidemiology, School of Public Health, Kyoto University Graduate School of Medicine, Kyoto, Japan; 3 Center for Innovative Research for Communities and Clinical Excellence, Fukushima Medical University, Fukushima, Japan; 4 Division of Nephrology, Showa University School of Medicine, Tokyo, Japan; 5 Division of Nephrology, Endocrinology and Metabolism, Tokai University School of Medicine, Isehara, Japan

**Keywords:** Epidemiology, Haemodialysis

## Abstract

Achieving calcium, phosphorus, and intact parathyroid hormone (PTH) targets helps improve dialysis population outcomes. We aimed to assess the population-level associations of achievement of those targets with survival using population-attributable-fractions (PAFs). We conducted a case-cohort study using data from 8229 haemodialysis patients with mildly elevated intact PTH, treated at 86 dialysis facilities in Japan. We examined associations among calcium, phosphorus, intact PTH, and mortality over 3 years. We estimated PAFs for achieving the targets of calcium, phosphorus, and intact PTH from the adjusted hazard ratios by Cox regression models. Proportions within the recommended range were 55.8%, 63.3%, and 39.1% for calcium (8.4–10.0 mg/dL), phosphorus (3.5–6.0 mg/dL) and intact PTH (60–240 pg/mL), respectively. The mortality rate was 5.7 per 100 person-years. Mortality was independently associated with non-achievement of targets. Regarding the population-level impact, statistically significant PAFs were found for achieving the combination of calcium and phosphorus (8.8%; 95% CI, 1.1–16.0). Further, PAF for combined calcium, phosphorus, and intact PTH was the largest (16.8%; 95% CI, 5.6–30.4). In conclusion, there might be additive and substantial population-level associations between survival and the achievement of calcium, phosphorus, and intact-PTH targets in the haemodialysis population with mildly elevated intact PTH.

## Introduction

Achieving calcium, phosphorus, and intact parathyroid hormone (PTH) targets is regarded as a core practice for improving outcomes in dialysis patients. Abnormal levels of calcium, phosphorus, and intact PTH are associated with higher mortality in these patients^1–3^. Therefore, clinical guidelines include target values for these bone-mineral markers^4–6^. To assess practice variations and outcomes in dialysis patients with mildly elevated intact-PTH levels, we conducted the Mineral and Bone Disorder Outcomes Study for Japanese Chronic Kidney Disease Stage 5D Patients (MBD-5D)^2,3,7,8^.

To achieve population-level improvements in dialysis outcomes, policy makers in various positions (such as physicians responsible for dialysis facilities, researchers responsible for developing clinical guidelines, and administrators responsible for health policies or medical insurance) seek evidence indicating how many lives could be saved if the management of dialysis patients were more successful. A few studies have examined the population-level impact of the management of bone-mineral markers in the dialysis population^9^ using epidemiological measures of population-attributable fractions (PAFs)^10,11^. PAFs indicate the percentage of undesirable outcomes that can be prevented by eliminating the risk factor from the population. In the present context, “successful” management refers to management that results in prescribed targets being achieved. A previous study using the Fresenius Medical Care North America Patient Statistical Profile system reported that 17.5% of deaths could be prevented by achieving target values of bone-mineral markers (calcium < 10 mg/dL, phosphorus < 5 mg/dL, and intact PTH < 600 pg/mL)^9^. However, PAFs depend on the prevalence of the risk factor and the strength of the association between the risk factor and the outcome, both of which are greatly affected by population characteristics and by the definition of the risk factor^11,12^. Practice patterns for treating dialysis patients with mineral and bone diseases have changed over time, especially after the introduction of cinacalcet^2,13^. An updated population-level approach to improving outcomes for dialysis patients requires information about the results of achieving the targets prescribed in clinical guidelines, so that those guidelines are more likely to be followed in facility-level practices. Thus, we need to re-evaluate the population-level impact of successful management of calcium, phosphorus, and intact PTH. Accordingly, with all-cause mortality as the outcome, we examined PAFs of successful management of bone-mineral markers in haemodialysis patients with mildly elevated intact-PTH levels, using data from the MBD-5D study.

## Results

### Patient characteristics

Among 8229 dialysis patients with mildly elevated intact-PTH levels in the whole cohort, we analysed 3276 (including 506 cases) in the subcohort and 720 cases outside the subcohort.

In the subcohort, the median age was 63 years, 38.5% were female, the median dialysis duration was 8.3 years, and 31.3% had diabetes. The median values for corrected calcium, phosphorus, and intact PTH were 9.7 mg/dL, 5.5 mg/dL, and 265 pg/mL, respectively. The proportion of patients who achieved bone-mineral marker targets^4^ were 55.8% for calcium (8.4–10.0 mg/dL), 63.3% for phosphorus (3.5–6.0 mg/dL), and 39.1% for intact PTH (60–240 pg/mL) (Table 1).

**Table 1:** Baseline patient characteristics.

	Whole cohort (n = 3,276)
Age (years)	63 (54–71)
Female	1,261 (38.5)
Dialysis duration (years)	8.3 (3.7–14.3)
Serum albumin (mg/dL)	3.8 (3.5–4.0)
Cardiovascular disease	1,965 (60.0)
Hypertension	2,613 (79.8)
Diabetes	1,026 (31.3)
Cancer	165 (5.0)
Single-pool Kt/V	1.41 (1.23–1.58)
Body mass index (kg/m^2^)	20.9 (19.0–23.3)
Corrected calcium (mg/dL)	9.7 (9.1–10.4)
Corrected calcium 8.4–10 mg/dL^a^	1,829 (55.8)
Phosphorus (mg/dL)	5.5 (4.6–6.3)
Phosphorus 3.5–6 mg/dL^a^	2,073 (63.3)
Intact PTH (pg/mL)	265 (195–392)
Intact PTH 60–240 pg/mL^a^	1,282 (39.1)
Cinacalcet use during the follow-up	1,384 (42.3)
Intravenous Vitamin D use at baseline	1,596 (48.7)
Phosphate binder use at baseline	2,995 (85.3)

Values are presented as median (range) or n (%).

^a^The targets of bone mineral markers used here were those outlined in the Japanese clinical guidelines.

PTH, parathyroid hormone.

### Associations between bone-mineral markers and mortality

We recorded 506 all-cause deaths that occurred during the follow-up of 8913 person-years for the subcohort and 720 all-cause deaths for those outside the subcohort. The all-cause mortality rate for the subcohort was 5.7 (95% confidence interval [CI], 5.2–6.2) per 100 person-years.

Table 2 and Supplementary Fig. 1 show a U-shaped association between corrected calcium and all-cause mortality after adjustment for potential confounders. Calcium levels of <8 mg/dL and ≥9 mg/dL were associated with higher mortality rates compared with calcium levels of 8–8.9 mg/dL. In the subcohort, only 2.2% of the patients had a calcium level < 8 mg/dL, and 80% of them had a calcium level ≥ 9 mg/dL.

**Table 2:** Association of mortality with calcium, phosphorus and intact PTH.

	n (%)	Hazard ratio (95% CI)
Calcium (mg/dL)		
<8	72 (2.2)	2.22 (1.43–3.44)
8–8.9	584 (17.8)	Reference
9–9.9	1,264 (38.6)	1.34 (1.08–1.66)
10–10.9	1,033 (31.5)	1.34 (1.04–1.71)
11–11.9	274 (8.4)	1.65 (1.23–2.20)
12+	49 (1.5)	2.32 (1.36–3.96)
Phosphorus (mg/dL)		
<3	66 (2.0)	1.31 (0.84–2.03)
3–3.9	277 (8.5)	1.07 (0.82–1.39)
4–4.9	772 (23.6)	Reference
5–5.9	1,017 (31.0)	1.21 (0.997–1.46)
6–6.9	707 (21.6)	1.23 (0.995–1.51)
7+	437 (13.3)	1.51 (1.18–1.94)
Intact PTH (pg/mL)		
<100	228 (7.0)	0.83 (0.62–1.13)
100–199	653 (19.9)	1.16 (0.96–1.40)
200–299	1,067 (32.6)	Reference
300–399	544 (16.6)	1.14 (0.93–1.40)
400–499	280 (8.6)	1.27 (0.97–1.67)
500–599	163 (5.0)	1.33 (0.96–1.84)
600–699	97 (3.0)	1.10 (0.70–1.73)
700+	244 (7.5)	1.50 (1.14–1.96)

Adjusted for age, sex, dialysis duration, serum albumin, cardiovascular disease, hypertension, diabetes mellitus, cancer, single-pool Kt/V and body mass index.

CI, confidence interval; PTH, parathyroid hormone.

Table 2 shows that phosphorus levels ≥ 7 mg/dL were associated with higher mortality rates compared to phosphorus levels of 4–4.9 mg/dL. In the subcohort, 13.3% of the patients had a phosphorus level ≥ 7 mg/dL.

Table 2 shows that intact-PTH levels ≥ 700 pg/mL were associated with higher mortality rates compared with intact PTH levels of 200–299 pg/mL. In the subcohort, 7.5% of the patients had intact PTH levels ≥ 700 pg/mL.

In a sensitivity analyse with adjustment for MBD-related medications by stratification based on cinacalcet use, we found similar associations between calcium, phosphorus, and mortality. Regarding intact-PTH levels, we did not find significant associations between high intact-PTH levels and mortality in the stratum of cinacalcet users.

### PAFs of bone-mineral markers

PAFs of each bone-mineral marker according to different cutoff values are shown in Supplementary Fig. 2. The PAFs varied by the cutoff values of the markers, as follows. Calcium: if a relatively low upper limit and thus a relatively narrow target-calcium range (8.4–10 mg/dL) was used, then the PAF was 4.9%. In contrast, if a higher upper limit and thus a wider target-calcium range (8.4–12 mg/dL) was used, then the PAF was lower, at only 1.4%. Similarly, for phosphorus, with a relatively low upper limit and thus a relatively narrow target-phosphorus range (3.5–5.0 mg/dL), the PAF was 5.1%, but with a higher upper limit and thus a wider target-phosphorus range (3.5–7 mg/dL), the PAF was lower at 2.3%. The same pattern was also seen with intact PTH. With a relatively low upper limit and thus a relatively narrow range of the intact-PTH target (60–180 pg/dL), the PAF was 8.8%, but with a higher upper limit and thus a wider range of the intact-PTH target (60–500 mg/dL), the PAF was only 0.9%.

**Figure 1:**
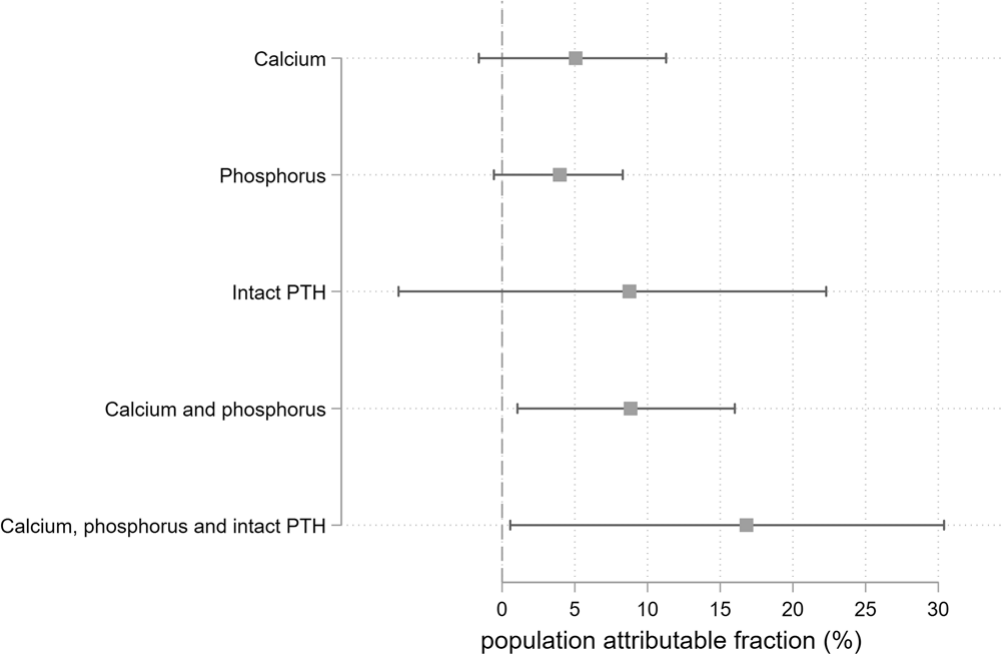
Population-attributable fractions (PAFs) for individual bone-mineral markers and their combinations (pairs). We computed the PAFs for the three bone-mineral markers individually and their combinations (pairs): calcium (8.4–10.0 mg/dL), phosphorus (3.5–6.0 mg/dL), and intact parathyroid hormone (60–240 pg/mL).

Figure 1 shows PAFs for the combinations of calcium, phosphorus, and intact PTH. PAFs were statistically significant when intact-PTH target levels of 60–180 pg/mL were achieved (8.8%; 95% CI, from −0.1 to 22.3). When calcium target levels (8.4–10 mg/dL) or phosphorus target levels (3.5–6 mg/dL) were achieved separately, the PAFs were not statistically significant; however, for the combination of calcium and phosphorus the PAF was statistically significant (8.8%; 95% CI, from 1.1 to 16.0). The highest PAF was for the combination of calcium, phosphorus, and intact PTH (16.8%; 95% CI, from 0.6 to 30.4).

Because the cut-off value of a risk factor may affect the PAF, we also estimated PAFs using different cut-off values of the markers (Supplementary Fig. S2). The PAFs estimated using lower cut-off values (MBD marker targets that were more difficult to attain) were associated with higher PAFs.

## Discussion and Conclusion

In dialysis patients with mildly elevated intact-PTH levels, mortality was independently associated with calcium, phosphorus, and intact PTH. The “shapes” of those associations are clearly evident from the analyses of continuous variables with restricted cubic splines, while their magnitudes are shown in the results of the analyses of ordered categorical variables. The all-cause mortality rate was 5.7 per 100 person-years. Regarding population-level associations of bone-mineral markers with outcomes, 8.8% of deaths could be prevented by achieving the guideline-mandated targets for calcium (from 8.4 to 10 mg/dL) and phosphorus (from 3.5 to 6 mg/dL) simultaneously. Furthermore, 16.8% of deaths might be prevented by achieving the guideline-mandated targets of calcium, phosphorus, and intact PTH (from 60 to180 pg/mL) simultaneously.

The population-level associations can be determined by the prevalence of a risk factor in the population and the strength of the association between that risk factor and a given outcome. First, we confirmed that mortality was associated with calcium, phosphorus, and intact PTH, as has been shown in previous studies^1,3,9^. Then, we estimated PAFs using different cutoff values for those markers, because cutoff values can be chosen arbitrarily and because the cutoff value can greatly affect the PAF. Supplementary Fig. 1 shows nonlinear associations between the three markers and mortality, and the distribution of the markers. We found that when narrow target ranges were used (i.e., when the upper cutoff values were relatively low) the PAFs were high. This is to be expected, because of the high prevalence of the risk factor and the strong association between the risk factor and the outcome. In general, to appropriately interpret the PAF as an index of population-level impact, one must carefully consider how the cutoff value of the risk factor was chosen^9,14^. If we assess the population-level impact by using PAFs appropriately, we can wisely choose guidelines and policies to improve population-level outcomes. Those “guidelines and policies” include not only the targets of markers as written in clinical guidelines, but also a facility’s treatment policy, and the coverage of treatments by medical insurance^10,11^.

Our results support the claim that an effective way to reduce mortality in this setting is to simultaneously achieve calcium, phosphorus, and intact-PTH targets. However, in some cases, treatments for abnormalities of the various markers may be incompatible. For example, a calcium-based phosphate binder for the treatment of high phosphorus may cause calcium overload, and a vitamin-D receptor activator for the treatment of high intact-PTH levels may cause over-absorption of calcium and phosphorus. We observed that combining cinacalcet with a vitamin-D receptor activator might help decrease intact-PTH levels and achieve calcium and phosphorus targets simultaneously^2^.

This study had several strengths. First, we performed an efficient analysis using case-cohort data. Taking advantage of the case-cohort design, we obtained detailed data only from patients in the subcohort and from cases outside the subcohort. Furthermore, there were no missing data for bone-mineral markers, all-cause mortality, or covariates (potential confounders). We were able to record data from all patients until the end of the follow-up period or death, whichever came first. Second, considering the effects of arbitrarily chosen cutoff values, we assessed various different approaches to define the risk factors of bone-mineral markers. We confirmed the previously-reported associations between bone-mineral markers and mortality, and we treated the variables both as ordered categorical variables and as continuous variables with restricted cubic splines. We also assessed the effects of various different cutoff values on PAFs.

This study also had some limitations. First, PAFs depend on the strong assumption of a causal relationship between the risk factor and the outcome^10,11^, which is always difficult to assess in observational studies^15^. Although we adjusted for many potential confounders in the Cox regression models, used to estimate adjusted PAFs, there might have been unmeasured confounders that affected the associations. However, the associations between bone-mineral markers and outcomes in this study were consistent with previous studies. Evidence of the causal effects of controlling bone-mineral markers on mortality will be needed from future intervention studies. To put it simply, the PAFs in this study were estimated from the associations found in an observational study, and we cannot infer the existence cause-effect relationships with regard to interventions and outcomes. Second, all of the data came from dialysis patients in Japan who had mildly elevated intact-PTH levels. These patients were relatively old (median age, 63 years) and had relatively long dialysis durations (median, 8.3 years), which was to be expected as it takes time to meet the criteria for mildly elevated intact-PTH levels. The one-year all-cause mortality proportion for the subcohort was 5.7% and was lower than that for all Japanese dialysis population reported as 9.2–10.2% by the Japanese Society for Dialysis Therapy^16^. This difference may be because our subcohort did not include incident dialysis populations and the MBD-5D study was conducted at relatively large dialysis facilities with more than 100 dialysis patients. Therefore, caution should be taken when applying our results to patients outside Japan, to younger patients, and to incident dialysis populations. Third, we assessed the population-level associations using fixed baseline values of bone-mineral markers, although those markers may have a time-varying nature. Because time-varying variables are suitable for analysing short-term and baseline variables are suitable for analysing long-term relationships, we selected fixed baseline values of bone-mineral markers to assess their association with mortality. In addition, the goal of estimating PAFs requires us to define the prevalence of risk factors at baseline. Since time-varying MBD-related medications are endogenous variables (i.e., they depend on patients’ conditions), adjusting for them in a time-dependent Cox model may cause bias. Therefore, we believe that a baseline Cox model is more suitable for our analysis. Fourth, the various risk factor (abnormalities in phosphorus, calcium, and intact PTH) may be associated with each other. The elimination of one risk factor could affect the distribution of the other risk factors. Therefore, we think that we should be cautious about the interpretation of differences in the PAFs between risk factors. Although it may be difficult to discuss the order of priorities set for clinical management of MBD markers, our results may support the recommendation to treat multiple risk factors (achieving calcium, phosphorus, and intact-PTH targets simultaneously). Finally, the MBD-5D selected patients as having a mildly elevated intact-PTH level if their intact PTH level was at least 180 pg/mL or they were receiving a vitamin-D receptor activator. The cut-off value of intact PTH in Japan was lower than in other countries. However, results from the population with moderately high intact-PTH levels may be also useful for other countries.

In conclusion, we confirmed the independent association of mortality with calcium, phosphorus, and intact-PTH levels in dialysis patients with mildly elevated intact-PTH levels. There might be an additive, substantial population-level associations between survival and achievement of calcium, phosphorus, and intact-PTH targets simultaneously.

## Materials and Methods

### Study cohort

We used data from the Mineral and Bone Disorder Outcomes Study for Japanese Chronic Kidney Disease Stage 5D Patients (MBD-5D) conducted between January 2008 and December 2010 (3-year follow-up). The MBD-5D is a case-cohort study involving 8229 patients in the whole cohort and 3276 patients in the subcohort, who were randomly selected from the whole cohort. Detailed data on patient characteristics were obtained from those in the subcohort and from cases (patients who died during follow-up) outside the subcohort. The MBD-5D included prevalent dialysis patients with mildly elevated intact-PTH levels who were treated at 86 dialysis facilities in Japan. Each facility had more than 100 patients. In the MBD-5D, patients were classified as having a mildly elevated intact-PTH level if their intact PTH level was at least 180 pg/mL or they were receiving a vitamin-D receptor activator. Details of the design of the MBD-5D have been described in a previous publication^7^.

In this study, we analysed data from 8229 haemodialysis patients with mildly elevated intact-PTH levels (the whole cohort).

### Exposures, outcomes, and covariates

The three bone-mineral markers of interest, calcium, phosphorus, and intact PTH, were measured in January 2008 (baseline). Calcium values were corrected if the albumin concentration was less than 4 mg/dL, according to the following equation: corrected calcium = calcium + 4 – albumin^4,17^. Whole PTH was measured with a third-generation PTH assay (immunoradiometric assay; reference, 9–39 pg/mL) at 13 of the 86 facilities, and were converted to intact-PTH values using the following equation: intact PTH (pg/mL) = 1.7 × whole PTH (pg/mL)^18,19^. The outcome was all-cause mortality during January 2008 to December 2010 (3-year follow-up). To adjust for confounding patient characteristic factors, we used age, sex (male or female), dialysis duration, serum albumin, cardiovascular disease (yes or no), hypertension (yes or no), diabetes mellitus (yes or no), cancer (yes or no), single-pool Kt/V, and body mass index as covariates.

### Ethical approval and informed consent

The study protocol was approved by the Central Ethics Committee at Kobe University School of Medicine (no. 754), and the requirement for informed consent was waived because we only used existing clinical data. The MBD-5D was conducted in accordance with the Declaration of Helsinki.

### Statistical analysis

For continuous variables, we estimated medians and interquartile ranges, for categorical variables, we estimated proportions. To examine nonlinear associations of mortality with calcium, phosphorus, and intact PTH, we developed multivariable Cox regression models using restricted cubic splines with five knots (at percentiles of 5, 27.5, 50, 72.5, and 95)^20^ after adjusting for potential confounders. The reference levels of calcium, phosphorus, and intact PTH were set to 9 mg/dL, 5 mg/dL, and 200 pg/mL, respectively. Given that we analysed case-cohort data^21^ of patients from the subcohort and cases outside the subcohort, we considered sampling weights (Borgan II weights)^22,23^ in our Cox models. Weights for cases were 1, and weights for subcohort noncases were calculated as follows: weight = (number of subcohort noncases)/(number of whole cohort noncases).

To estimate the population-level impact of achieving the calcium, phosphorus, and intact-PTH targets, we estimated PAFs from adjusted hazard ratios determined by Cox regression models^24,25^. PAFs indicated the proportion of cases (i.e., deaths) that could be prevented by eliminating the risk factor (i.e., abnormal calcium, phosphorus, and intact-PTH levels) from the population. First, we calculated adjusted hazard ratios (HRs) for the association between each risk factor and mortality using Cox regression models and adjusting for potential confounders. Then we calculated the proportions of patients with those risk factors. Finally, we estimated PAFs using those proportions and HRs. Because the proportion of patients with the risk factor and the association between the risk factor and the outcome depend on the definition of the risk factor, we calculated PAFs using different cutoff values for the markers. The lower cutoff values were set to 8.4 mg/dL for calcium, 3.5 mg/dL for phosphorus, and 60 pg/mL for intact PTH. The upper cutoff values were set to 9–13 mg/dL (per 1 mg/dL) for calcium, 5–8 mg/dL (per 1 mg/dL) for phosphorus, and 100–1000 pg/mL (per 100 pg/mL) for intact PTH.

We also estimated PAFs for combinations of the markers. The targets we used were defined in the clinical guidelines of the Japanese Society for Dialysis Therapy (JSDT)^4^: calcium (8.4–10.0 mg/dL), phosphorus (3.5–6.0 mg/dL), and intact PTH (60–240 pg/mL). Then, we calculated PAFs for achievement of the combined targets.

In a sensitivity analysis to examine the associations between MBD markers and mortality with adjustment for MBD-related medications, we adjusted for intravenous Vitamin D use and phosphate-binder use, after stratification by cinacalcet use.

All analyses were performed using Stata version 15.1 (StataCorp, College Station, TX, USA). All tests were two-sided, and p < 0.05 was considered statistically significant.

## Supplementary information

Supplementary Information
