# Supplementary material for: Population-level associations of achievement of targets for bone-mineral markers with survival in haemodialysis patients with mildly elevated intact-PTH levels: a case-cohort study
Source: Sci Rep. 2019 Aug 5;9:11301. doi: 10.1038/s41598-019-47852-8 (PMC6683189; doi:10.1038/s41598-019-47852-8)
Supplement: Supplementary file 1 — Supplementary Information [file 41598_2019_47852_MOESM1_ESM.pdf]

Population-level associations of achievement of targets for bone-mineral markers with survival in haemodialysis patients with mildly elevated intact-PTH levels: a case-cohort study

Shingo Fukuma, Shunichi Fukuhara, Sayaka Shimizu, Tadao Akizawa, Masafumi Fukagawa

Supplementary Information

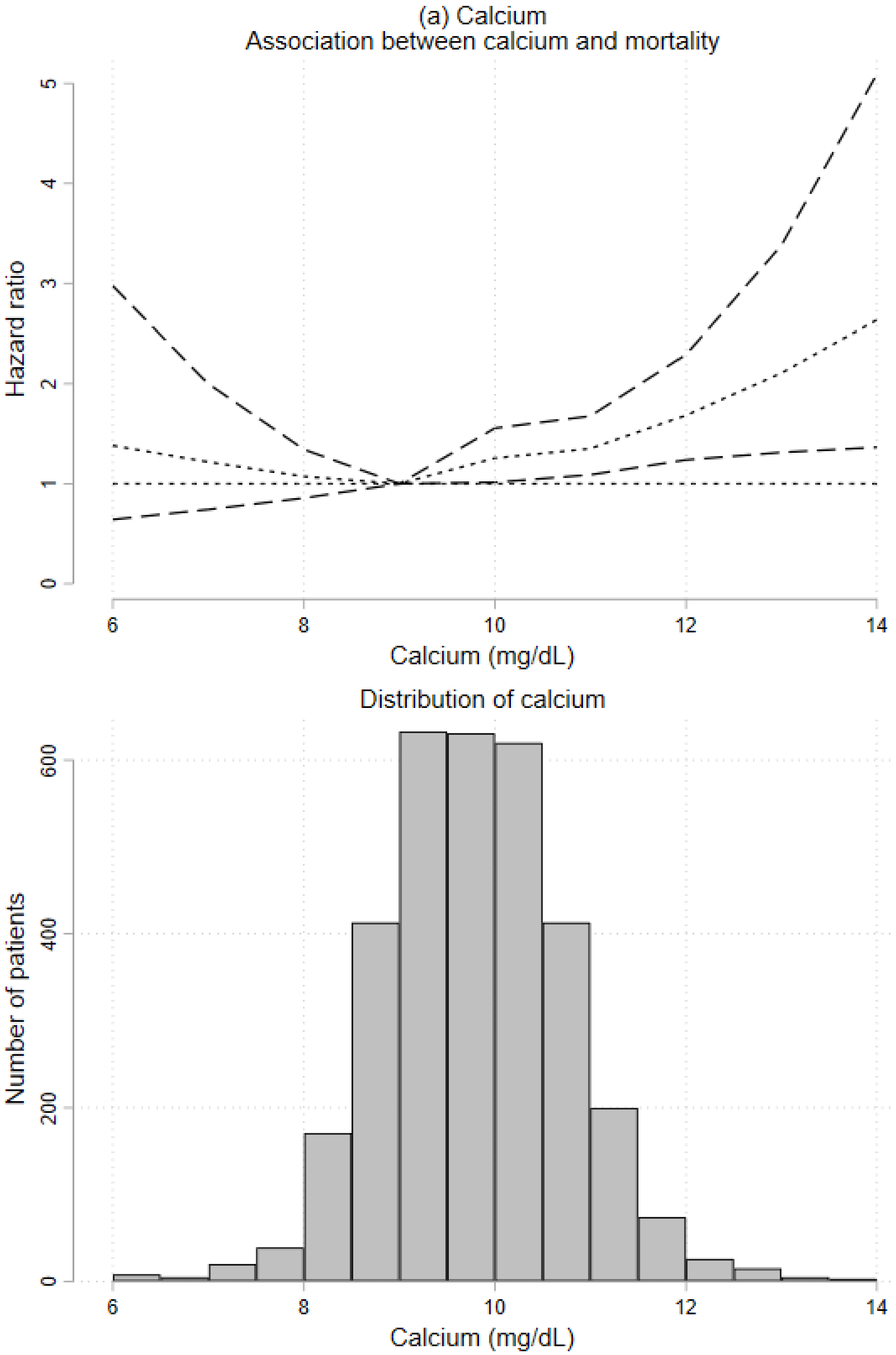
(a) Calcium
Association between calcium and mortality
Hazard ratio
0
1
2
3
4
5
6
8
10
12
14
Calcium (mg/dL)
Distribution of calcium
Number of patients
0
200
400
600
6
8
10
12
14
Calcium (mg/dL)

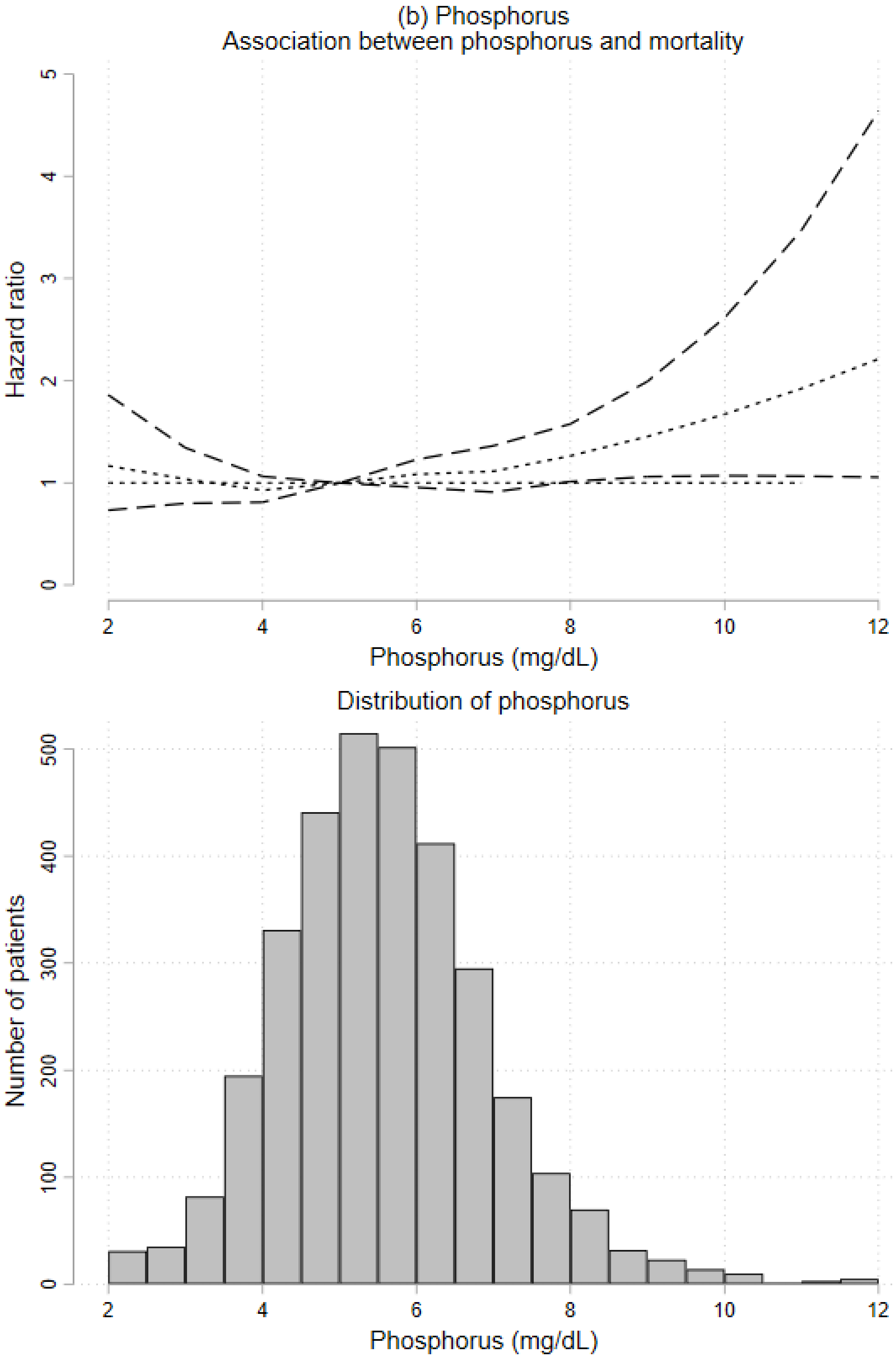
(b) Phosphorus
Association between phosphorus and mortality
Hazard ratio
0
1
2
3
4
5
2
4
6
8
10
12
Phosphorus (mg/dL)
Distribution of phosphorus
Number of patients
0
100
200
300
400
500
2
4
6
8
10
12
Phosphorus (mg/dL)

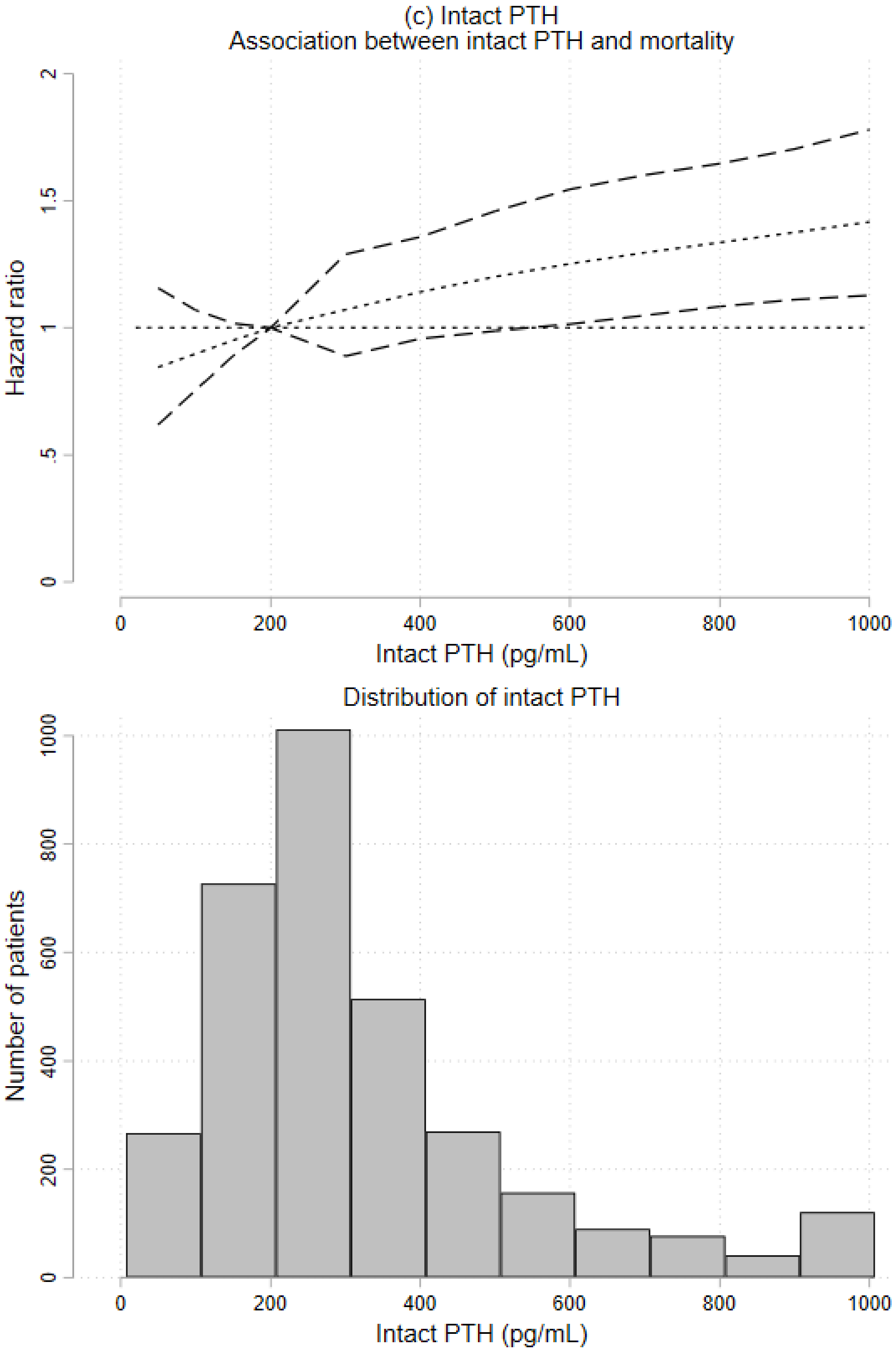
(c) Intact PTH
Association between intact PTH and mortality
Hazard ratio
0
.5
1
1.5
2
0
200
400
600
800
1000
Intact PTH (pg/mL)
Distribution of intact PTH
Number of patients
0
200
400
600
800
1000
0
200
400
600
800
1000
Intact PTH (pg/mL)

Supplementary Figure S1. Nonlinear associations between bone mineral markers and all-cause mortality and distribution of bone mineral markers: (a) calcium, (b) phosphorus and (c) intact parathyroid hormone (PTH). For the nonlinear association, we developed multivariable Cox regression models using restricted cubic splines with five knots (at percentiles 5, 27.5, 50th, 72.5 and 95) after adjusting for other bone mineral markers, age, sex, dialysis duration, serum albumin, cardiovascular disease, hypertension, diabetes mellitus, cancer, single-pool Kt/V and body mass index.

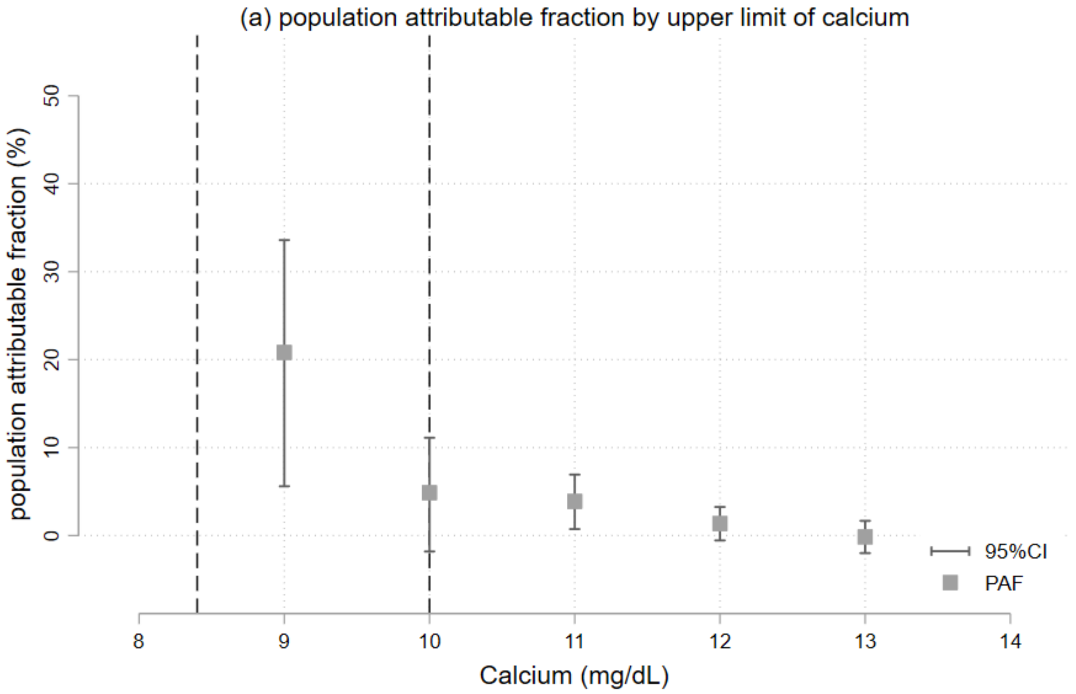
(a) population attributable fraction by upper limit of calcium
population attributable fraction (%)
0
10
20
30
40
50
8
9
10
11
12
13
14
Calcium (mg/dL)
95%CI
PAF

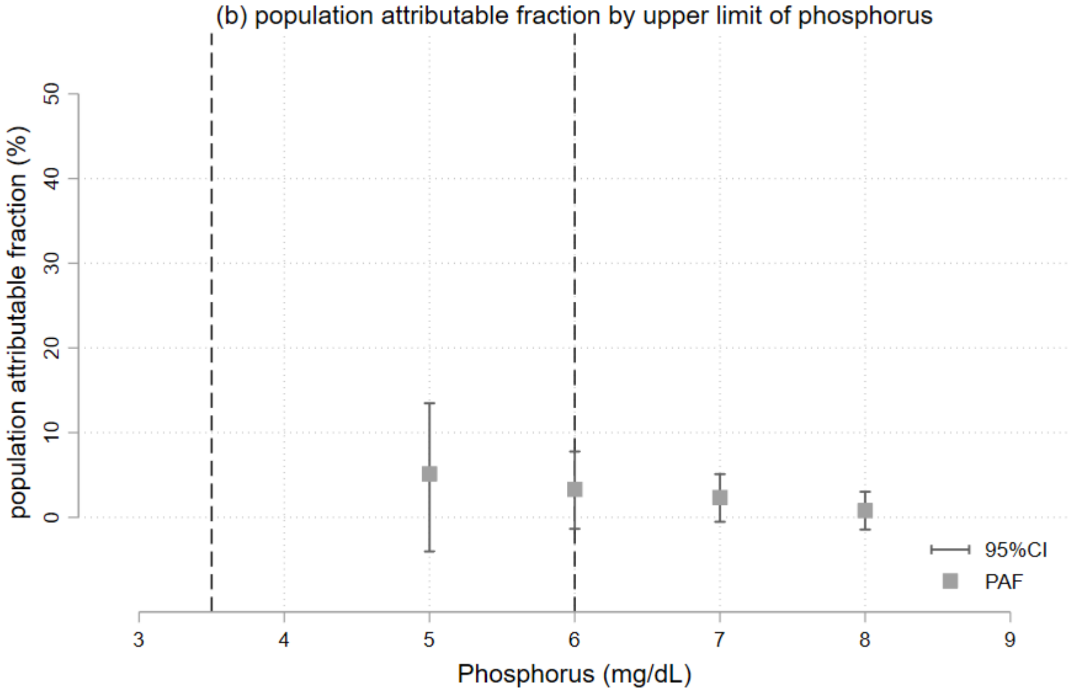
(b) population attributable fraction by upper limit of phosphorus
population attributable fraction (%)
0
10
20
30
40
50
3
4
5
6
7
8
9
Phosphorus (mg/dL)
95%CI
PAF

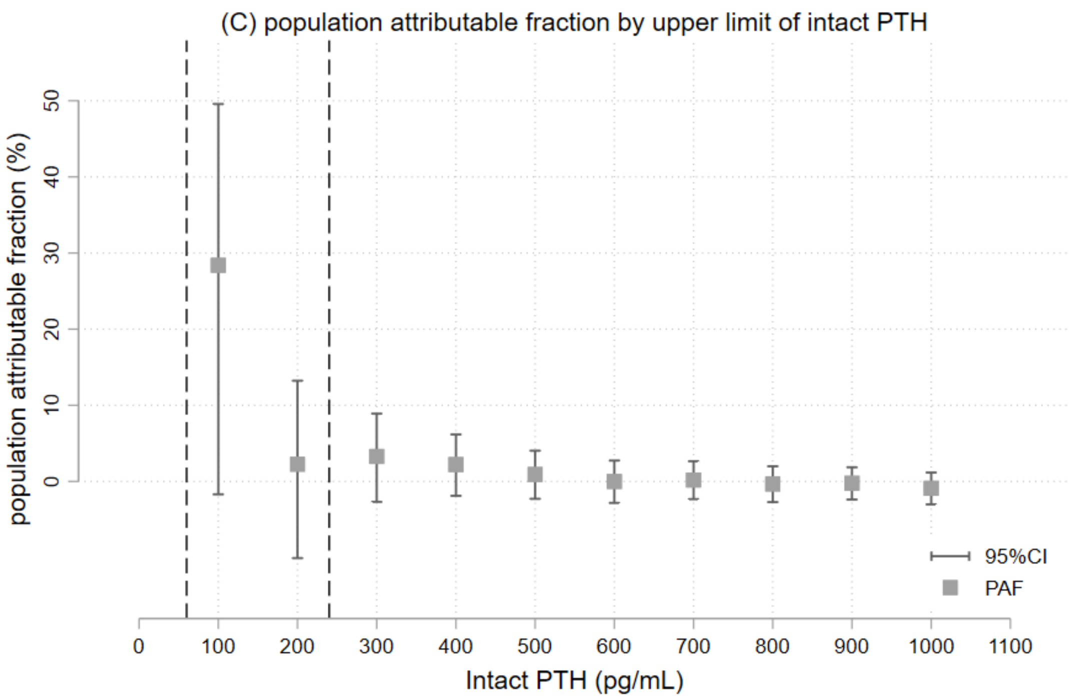

Supplementary Figure S2. Population-attributable fractions (PAFs) for bone mineral markers by different upper cutoff values. To estimate the population-level impact of (a) calcium, (b) phosphorus and (c) intact parathyroid hormone, we computed the PAFs using different cutoff values for those bone mineral markers.

Supplemental Table 1. Association of mortality with calcium, phosphorus, and intact PTH, with adjustment for MBD-related medications by stratification based on cinacalcet use

	Cinacalcet non-user<br>N=2,611<br>Hazard ratio (95% CI)	Cinacalcet user<br>N=1,384<br>Hazard ratio (95% CI)
Calcium (mg/dL)		
<8	2.19 (1.41-3.40)	2.67 (0.24-30.43)
8-8.9	Reference	Reference
9-9.9	1.59 (1.28-1.98)	1.65 (0.45-6.07)
10-10.9	2.17 (1.66-2.83)	2.25 (0.63-8.03)
11-11.9	2.87 (2.03-4.04)	3.54 (0.92-13.63)
12+	4.95 (2.89-8.48)	2.58 (0.47-14.29)
Phosphorus (mg/dL)		
<3	1.17 (0.77-1.80)	1.57 (0.31-8.00)
3-3.9	0.96 (0.73-1.27)	1.43 (0.58-3.50)
4-4.9	Reference	Reference
5-5.9	1.21 (0.99-1.48)	1.61 (0.93-2.80)
6-6.9	1.28 (1.02-1.62)	1.05 (0.55-2.01)
7+	1.79 (1.37-2.35)	1.59 (0.81-3.11)
Intact PTH (pg/mL)		
<100	0.71 (0.53-0.98)	0.21 (0.03-1.60)
100-199	1.05 (0.85-1.28)	0.96 (0.53-1.76)

200-299	Reference	Reference
300-399	1.34 (1.07-1.67)	0.59 (0.31-1.11)
400-499	1.70 (1.24-2.32)	0.84 (0.44-1.64)
500-599	1.42 (0.96-2.09)	1.62 (0.82-3.18)
600-699	1.90 (1.14-3.16)	0.70 (0.23-2.14)
700+	1.98 (1.43-2.74)	1.24 (0.66-2.33)

Adjusted for age, sex, dialysis duration, serum albumin, cardiovascular disease, hypertension, diabetes mellitus, cancer, single-pool Kt/V, body mass index, intravenous Vitamin D use, and phosphate binder use.

CI, confidence interval; PTH, parathyroid hormone.
